# A pilot transcriptomic study of a novel multitargeted BRT regimen for anti–MDA5 antibody-positive dermatomyositis: improving survival over conventional therapy

**DOI:** 10.3389/fimmu.2025.1568338

**Published:** 2025-08-07

**Authors:** Moe Tokunaga, Yu Nakai, Yoshiharu Sato, Mitori Hiratsuka, Yoshinori Matsumoto, Takeshi Nakatsue, Takako Saeki, Takatsune Umayahara, Jun Wada, Yoshinobu Koyama

**Affiliations:** ^1^ Division of Rheumatology, Center for Autoimmune Diseases, Japanese Red Cross Okayama Hospital, Okayama, Japan; ^2^ Department of Nephrology, Rheumatology, Endocrinology and Metabolism, Okayama University Graduate School of Medicine, Dentistry and Pharmaceutical Sciences, Okayama, Japan; ^3^ DNA Chip Research Inc., Medical Laboratory, Kawasaki, Japan; ^4^ Division of Rheumatology and Nephrology, Department of Internal Medicine, Nagaoka Red Cross Hospital, Niigata, Japan; ^5^ Division of Dermatology, Center for Autoimmune Diseases, Japanese Red Cross Okayama Hospital, Okayama, Japan

**Keywords:** anti-MDA5 antibody-positive dermatomyositis (MDA5-DM), JAK inhibitor, baricitinib, rituximab, multitargeted treatment, IFN signature, transcriptome analysis

## Abstract

**Background:**

Anti-melanoma differentiation-associated gene 5 antibody-positive dermatomyositis (MDA5-DM) is associated with severe outcomes, primarily due to rapidly progressive interstitial lung disease (RP-ILD), which is often refractory to standard therapies such as calcineurin inhibitors (e.g., tacrolimus) combined with cyclophosphamide (TC-Tx). This study evaluated the efficacy of a novel multitargeted regimen combining baricitinib, rituximab, and tacrolimus (BRT-Tx) in improving survival outcomes for MDA5-DM patients with poor prognostic factors.

**Methods:**

Fourteen MDA5-DM patients with multiple adverse prognostic factors were studied. Seven received the BRT-Tx regimen, and the remaining seven, previously treated with TC-Tx, served as historical controls. Twelve-month survival was assessed. Transcriptome analysis was performed for six patients (BRT=3, TC=3), beginning with cluster analysis to evaluate whether changes in peripheral blood gene expression varied according to treatment or prognosis. Gene ontology analysis characterized expression profiles in survivors and distinguished treatment effects. Alterations in the type I, II, and III interferon signatures were also assessed.

**Results:**

In the TC-Tx group, four of seven patients succumbed to RP-ILD, whereas all seven BRT-Tx patients survived the 12-month observation period. Only one BRT-Tx patient required combined rescue therapies, including plasma exchange, and one case of unexplained limbic encephalitis (LE) occurred. Cytomegalovirus reactivation was observed in both groups (BRT: 5/7; TC: 6/7). Transcriptomic analysis revealed no treatment-specific clustering of differentially expressed genes (DEGs) before and after therapy. However, survivors and nonsurvivors formed distinct clusters, with survivors showing significant posttreatment suppression of B-cell-related gene expression. Moreover, interferon signature scores were significantly lower after treatment in survivors than in nonsurvivors. BRT-Tx effectively suppressed B-cell-mediated immune responses and maintained a low interferon signature, while TC-Tx resulted in nonspecific gene suppression, and in nonsurvivors, an elevated interferon signature was observed.

**Conclusion:**

BRT-Tx has the potential to improve survival in MDA5-DM patients by effectively targeting hyperactive immune pathways. The combination of rituximab and tacrolimus is expected to disrupt B-cell–T-cell interactions and reduce autoantibody production, whereas baricitinib may suppress both IFN and GM-CSF signaling, regulating excessive autoimmunity mediated by cells such as macrophages. Unlike TC-Tx, BRT-Tx avoids cyclophosphamide-associated risks such as infertility and secondary malignancies. Future randomized controlled trials are warranted to validate its efficacy and safety.

## Introduction

Anti-melanoma differentiation-associated gene 5 (MDA5)-positive DM/CADM is known to be fatal when complicated by rapid progressive interstitial lung disease (RP-ILD), with a prevalence of 39–100% (1).

MDA5 is a cytoplasmic RNA virus sensor that belongs to the RIG-I-like receptor family (2). Upon activation, it transmits signals downstream, leading to the production of inflammatory cytokines such as type I IFN and inducing the death of infected cells and antigen-specific immune responses (3).

In 2005, Sato et al. identified antibodies specific to clinically amyopathic dermatomyositis (CADM), termed anti-CADM140 antibodies (4). Subsequent research revealed MDA5 as the CADM140 autoantigen, leading to its designation as the anti-MDA5 antibody (5, 6). However, the mechanisms underlying the production of antibodies against cytoplasmic MDA5 remain unclear.

MDA5 recognizes RNA from viruses (7), such as picornaviruses or coronaviruses (1). In the case of COVID-19, rapid RNA replication occurs in the epithelial cells of the lungs and skin, which is detected by MDA5, a cytoplasmic RNA virus sensor (8). Interestingly, chest HRCT findings and blood cytokine profiles in COVID-19 patients closely resemble those observed in MDA5-DM patients (9–11).

In typical viral infections, IFN serves as a defense mechanism against viral replication.

However, in cases of severe pneumonia caused by COVID-19 and the pathogenesis of MDA5-DM, an aberrantly amplified immune response, alongside dysregulated interferon (IFN) signaling pathways, is postulated to play a critical role. This pathological dysregulation not only intensifies inflammatory processes but also contributes to the autoimmune manifestations characteristic of these conditions (1). In a previous study, we conducted transcriptome analysis of peripheral blood samples obtained prior to treatment from twelve patients with anti-ARS antibody-positive dermatomyositis and seven with MDA5-DM. Hierarchical clustering analysis clearly delineated the two disease groups, with MDA5-DM patients exhibiting increased expression of genes related to innate immune responses, antiviral defense mechanisms, and Fcγ receptor–mediated phagocytosis. Among a subset of five MDA5-DM patients with matched pre- and posttreatment samples (three survivors and two nonsurvivors), a marked posttreatment downregulation of genes involved in antiviral immunity—including type I and II interferon signaling as well as RIG-I-like receptor pathways—was observed exclusively in survivors (12).

Several poor prognostic factors are known to be related to MDA5-DM, including the presence of ILDs (13), an anti-MDA5 antibody titer ≥500 U/L (14), ferritin ≥636 ng/mL, LDH ≥355 U/L, CRP ≥0.8 mg/dL, KL-6 ≥1000 IU/mL (15), age>50 (16) and anti-SSA/Ro52 antibody positivity (17, 18). Additionally, a cluster analysis of MDA5-DM patients (n=121) on the basis of clinical symptoms revealed three subgroups. Among these patients, 18.1% were classified into the poor prognostic group; these patients had a high probability (93.3%) of having rapidly progressive ILD, and their 3-month survival rate was only 20%. In a prognostic prediction model based on clinical manifestations, the absence of Raynaud’s phenomenon and arthritis has been identified as a strong predictor of poor prognosis, with patients lacking both symptoms exhibiting the highest likelihood of belonging to the most unfavorable prognostic group (19).

Recently, several treatment options for MDA5-DM patients with ILD have been reported. Initial treatment with TC-Tx [high-dose glucocorticoids (GCs), calcineurin inhibitors such as tacrolimus (TAC), and intravenous cyclophosphamide (CYC)] was shown to improve 6-month survival compared with that of patients receiving conventional stepwise therapy (89% *vs*. 33%, P<0.0001) (20). In 2019, treatment with the Janus kinase (JAK) inhibitor tofacitinib (TOF) resulted in a 100% survival rate at 6 months, compared with 78% with conventional treatment (21); however, another group later conducted a similar regimen with a 6-month survival rate of 61.5% (22). There are also reports of increased survival rates with the combination of rituximab (RTX) (23, 24) and plasma exchange (PE) (25, 26) in patients who are resistant to conventional therapy. There is no established treatment for this disease, and although several reports of outcomes with treatment regimens exist, no studies have targeted patients who are predicted to have a poor prognosis.

We administered a combination therapy comprising high-dose GC, the JAK1/2 inhibitor baricitinib (BAR), RTX, and TAC (BRT-Tx) to patients with MDA5-DM, which presented multiple poor prognostic factors. In this study, alongside historical cases treated with TC-Tx, we explored changes in peripheral blood gene expression to elucidate how these alterations varied between survivors and nonsurvivors as well as between the BRT and TC therapeutic approaches.

## Patients and methods

### Patients

Fourteen Japanese patients who were diagnosed with MDA5-DM at Okayama Red Cross Hospital and Nagaoka Red Cross Hospital were included in this retrospective study. Seven patients received BRT-Tx (after February 2021), while seven historical patients were assigned to the TC-Tx group (after June 2015). The diagnosis of DM was made according to the 2017 Eular/ACR classification criteria for idiopathic inflammatory myopathies (IIMs) (27), and MDA5-DM was diagnosed in patients with characteristic skin symptoms (heliotrope sign, Gottron sign, periungual erythema, etc.) and positive anti-MDA5 antibodies (28). ILD was detected via high-resolution computed tomography (HRCT).

BRT-Tx was administered to patients who met two or more of the following eight poor prognostic factors: 1) the presence of ILD on chest HRCT consistent with MDA5-DM-related lung lesions (mandatory criterion) (13), 2) anti-MDA5 antibody titer ≥500 Index (14), 3) ferritin ≥636 ng/mL (13), 4) LDH≥355 units/L (13), 5) CRP≥0.8 mg/dL (15), 6) KL-6≥1000 IU/mL (15), 7) age>50 years (16) and 8) positivity for anti-Ro-52/SS-A antibodies (17, 18).

The data from MDA5-DM patients treated with BRT therapy were compared with those from historical patients treated with TC-Tx (TC: n = 7, BRT: n = 7). Among these, six patients were included in the gene expression analysis (TC-1 (fatal), TC-2, TC-3 (fatal), BRT-1, BRT-2, and BRT-3). The observation period spanned 12 months, with survival rates assessed at 12 months following the initiation of treatment.

### BRT treatment regimen

High-dose GC: In principle, the treatment regimen includes administering 1000 mg/day of methylprednisolone (mPSL) for the first three days, beginning on day 1, followed by a transition to prednisolone (PSL) at a dosage of 1 mg/kg/day. The dose is then gradually reduced by 10–20% every 2–4 weeks on the basis of disease activity.

Baricitinib (BAR): BAR is typically initiated at a dose of 4 mg starting on day 1. If the estimated glomerular filtration rate (eGFR) is <60 ml/min, the starting dose is reduced to 2 mg. In cases where the ferritin concentration is <500 ng/mL and the disease is clinically controlled, the dose is halved, and treatment is discontinued upon normalization of ferritin levels. In instances of concomitant iron deficiency anemia, where ferritin levels may not reliably reflect the disease status, dose adjustments should be made on the basis of other clinical parameters, such as LDH or KL-6, via a comprehensive approach.

Rituximab (RTX): RTX is typically initiated on day 4, with a dose of 375mg/m^2^ per infusion once weekly for four consecutive weeks, followed by up to four additional infusions every 6 months, in principle. Subsequent dosing should be discontinued once a negative anti-MDA5 antibody status is confirmed.

Tacrolimus (TAC): The initial dose of TAC is 0.0375 mg/kg, which is administered orally twice daily. The target 12-hour trough blood concentration is subsequently maintained within the range of 5–10 ng/mL, with dose adjustments made on the basis of regular monitoring of the trough concentration (**Table 1**).

**Table 1:** Summary of BRT therapies.

BRT treatment regimen
1. **High-dose GC:** Generally, mPSL 1000 mg/day for 3 days starting from Day 1, followed by switching to PSL 1 mg/kg/day, and dose reduction by 10-20% every 2 to 4 weeks depending on disease activity.
2. **Baricitinib:** Generally, initiated at 4 mg from Day 1. If eGFR is <60, start at 2 mg. If ferritin is <500 ng/mL and the disease is controlled, halve the dose, and discontinue upon normalization of ferritin levels.
3. **Rituximab:** Generally, start on Day 4; 375 mg/m^2^ per infusion once weekly for four consecutive weeks, then up to 4 times every 6 months in principle. Subsequent doses should be discontinued after anti-MDA5 antibody-negative is confirmed.
4. **Tacrolimus:** Starting from Day 1, orally administer 0.0375 mg/kg/dose twice daily after morning and evening meals, adjusting to achieve a 12-hour trough concentration of 5 to 10 ng/mL.
Combined Rescue therapy
In the event of disease relapse after the initiation of treatment, it is recommended that the following interventions be employed in combination with BRT therapy: (1) administration of High-dose GC, (2) plasma exchange performed 5–7 times, followed by (3) intravenous immunoglobulin therapy (IVIG).
Prevention of adverse events
1. **Prophylactic administration:** Sulfamethoxazole-trimethoprim and antifungal agents should be used to prevent (PCP) and fungal infections. Prophylactic administration of acyclovir is also recommended for prophylaxis during baricitinib administration.
2. **Monitor reactivation of latent infection:** CMV reactivation should be monitored at least once every 1–2 weeks, especially while patients are receiving Bar, and If reactivation is detected, appropriate antiviral therapy should be administered.

### TC treatment regimen

High-dose GC: In principle, the treatment regimen includes the administration of 1000 mg/day mPSL for the first three days, beginning on day 1, followed by a transition to prednisolone (PSL) at a dosage of 1 mg/kg/day. The dose is then gradually reduced by 10–20% every 2–4 weeks on the basis of disease activity.

Cyclophosphamide (CYC): CYC is administered intravenously at a dose of 500 to 1000 mg/m², for a total of up to 10 times. Initially, it is given once every 2–4 weeks for six cycles, followed by administration every 4–8 weeks.

Tacrolimus (TAC): The initial dose of TAC is 0.0375 mg/kg, which is administered orally twice daily. The target 12-hour trough blood concentration is subsequently maintained within the range of 5–10 ng/mL, with dose adjustments made on the basis of regular monitoring of the trough concentration.

### Combined rescue treatment

During the course of treatment, if disease relapse was observed, adjuvant therapies, such as (1) mPSL pulse therapy or increased dosages of steroids (2), plasma exchange (PE) (5–7 sessions), and (3) intravenous immunoglobulin therapy (IVIG), were added.

### Statistical analysis

Statistical analyses were performed to evaluate differences in patient backgrounds between the TC-Tx and BRT-Tx groups. Statistical tests used include the Mann–Whitney U test, Fisher’s exact test, chi-square test, and log-rank test (for prognosis based on 1-year follow-up).

### Gene analysis: RNA extraction

Peripheral blood samples were collected at baseline and three months after treatment, or—if applicable—within two weeks prior to death (i.e., approximately 2 to 2.5 months posttreatment) via PAXgene^®^ Blood RNA tubes (Nippon Becton Dickinson, Tokyo, Japan). Total RNA was extracted via the PAXgene^®^ Blood miRNA Kit (with DNase treatment) (QIAGEN K.K., Tokyo, Japan) following the manufacturer’s protocol. The quantity and quality of the total RNA were assessed via the SureSelect Strand-Specific RNA Kit (Agilent Technologies, Inc., CA, USA).

### Next-generation sequencing

The RNA-Seq libraries were constructed via the Strand-Specific mRNA-Seq PolyA Kit (Agilent Technologies, Inc., CA, USA) according to the manufacturer’s protocol. For the constructed sequencing libraries, sequencing data were generated (75 bp single-end) via the NextSeq500 platform (Illumina, CA, USA). To process the sequencing reads, Illumina adapter sequences were removed via Trimmomatic v0.33. The trimmed reads were then mapped to the human genome (hg38) and reference transcripts via STAR v2.7.9. The reads aligned to the Ensembl v78 reference genes were quantified with Subread 2.0.3. The average number of reads per sample was set to approximately 20 million.

### Transcriptome analysis of the peripheral blood

Differentially expressed genes (DEGs) were defined as those exhibiting an absolute log_2_ fold change ≥ 1 and a *P*-value < 0.05 between pre- and posttreatment samples. These DEGs were subsequently subjected to clustering analysis (29) to determine whether their expression patterns were influenced primarily by differences in treatment regimens or by clinical outcomes, such as survival or death. Next, to investigate the gene expression changes associated with differences in treatment regimens or outcomes, we performed Gene Ontology (GO) analysis (30) on the genes that were downregulated after treatment among the DEGs. Additionally, gene set variation analysis (GSVA) (31) was performed to explore the characteristics of the DEGs that differed between the BRT and TC treatment groups.

### Interferon signatures

The IFN signature refers to the elevated gene expression induced by IFN. Among the IFN-stimulated genes (ISGs) associated with type 1 IFNs, 73 genes (gene set enrichment analysis (GSEA) C2 reactome dataset), type 2 IFN ISGs, 94 genes (GSEA C2 reactome dataset), and type 3 IFN ISGs, 10 genes (GSEA wikipathway gene set), were analyzed and scored (32).

For the scoring method, the gene expression of 6 patients (TC therapy: n=3, BRT therapy: n=3) before and 2–3 months after treatment was z scored (if the cohort/batch was different, the z score was obtained after correcting for batch by mean centering). A coefficient of +1 for high expression and -1 for low expression was assigned to each gene. The z score was multiplied by the coefficient and summed to obtain the IFN signature score.

## Results

### Characteristics of patients at baseline

**Table 2** shows the background characteristics of patients treated with TC (n=7) and patients treated with BRT (n=7). The number of risk factors was 4.43 ± 0.79 in the TC group and 4.57 ± 0.98 in the BRT group, with no significant difference. Welch’s t test revealed that age was significantly greater in the TC group than in the BRT group (TC: 65.4 ± 5.7; BRT: 48.6 ± 11.4; p = 0.015). In contrast, SP-D (human surfactant protein D) levels were significantly elevated in the BRT group relative to those in the TC group (TC: 33.0 ± 25.4; BRT: 83.8 ± 26.6; p = 0.038). Although not statistically significant, KL-6 levels tended to be higher in the BRT group (TC: 515.0 ± 196.1; BRT: 851.1 ± 346.6; p = 0.053). For qualitative data, a chi-square test was performed. No significant differences were observed in the other items.

**Table 2:** Baseline characteristics of the patients.

Variable	TC group	BRT group	p-value
† Prognosis	fatal: 4, alive: 3	fatal: 0, alive: 7	0.045
No. of risk factors	4.4 ± 0.8	4.6 ± 1.0	0.836
†*Age, years	65.4 ± 5.7	48.6 ± 11.4	0.015
Sex	F: 6, M: 1	F: 5, M: 2	1.000
*Ferritin, ng/mL	557.0 ± 501.3	1311.6 ± 1188.8	0.128
*LDH, U/L	369.9 ± 84.5	452.0 ± 235.5	0.805
AST, U/L	80.4 ± 75.7	92.4 ± 98.3	0.609
ALT, U/L	44.3 ± 38.9	87.4 ± 79.6	0.306
Creatinine, mg/dL	0.6 ± 0.1	0.6 ± 0.2	0.701
CK, U/L	353.9 ± 449.4	170.6 ± 243.4	0.306
*KL-6, U/mL	515.0 ± 196.1	851.1 ± 346.6	0.053
† SP-D, ng/mL	33.0 ± 25.4	83.8 ± 26.6	0.038
*CRP, mg/dL	1.6 ± 1.3	1.0 ± 1.5	0.209
ESR, mm/hr	54.2 ± 32.2	39.9 ± 11.2	0.391
*anti-MDA5 antibody, index	3157.9 ± 1360.6	2683.6 ± 2058.4	0.383
*SS-A/Ro-52 antibody positive, %	33.3	0.0	0.192
*ILD at baseline	-: 0, +: 4, ++: 2, +++: 1	-: 0, +: 5, ++: 1, +++: 1	0.801
Heliotrope rash, %	57.1	28.9	0.592
Gottron’s sign,%	100.0	100.0	1.000
Arthritis, %	28.6	28.6	1.000
Raynaud phenomenon, %	0.0	0.0	1.000
Lower limb muscle weakness, %	28.6	28.6	1.000

Comparison of baseline characteristics between the TC and BRT groups. Continuous variables are expressed as mean ± standard deviation. Categorical variables are presented as counts. Statistical tests used include the Mann–Whitney U test, Fisher’s exact test, chi-square test, and log-rank test (for prognosis based on 1-year follow-up).

*Asterisks indicate poor prognostic predictors. †Daggers indicate items with significant differences between treatment groups.

M, male; F, female; DM, dermatomyositis; CADM, clinically amyopathic dermatomyositis; CK, creatine kinase; LDH, lactate dehydrogenase; CRP, C-reactive protein; ESR, erythrocyte sedimentation rate; KL-6, Krebs von den Lungen 6; SP-D, surfactant protein D; anti–MDA5, anti–melanoma differentiation–associated gene 5; SS-A, Sjogren’s syndrome A antigen.

### Treatment course of each patient

In the TC-Tx group, the survival rate decreased progressively with the number of poor prognostic factors: 100% with three factors, 50% with four, and 25% with five. In contrast, the BRT-Tx group exhibited a consistent 100% survival rate regardless of the number of poor prognostic factors (ranging from three to six). This difference in survival trends between the two groups was statistically significant (log-rank test, *P* = 0.045), suggesting that BRT-Tx may offer superior survival benefits particularly in patients with high-risk features.

In the BRT group, patient BRT-1 had previously undergone two steroid pulse therapies prior to the initiation of BRT treatment. Despite these efforts, the disease remained completely uncontrolled, and the patient exhibited severe ILD complicated by mediastinal pneumothorax. The chest CT findings closely resembled those observed in severe COVID-19 pneumonia patients. For patient BRT-4, mild worsening of ILD and an increase in the anti-MDA5 antibody titer were observed three months after treatment initiation. In response, combined rescue therapy—including mPSL pulse therapy, PE, and IVIG—was administered, leading to recovery without recurrence. The remaining six BRT patients did not require combined rescue therapy.

No serious adverse events leading to death were observed in any of the patients. In the BRT-1 patient, however, fever and seizures developed on day 91, while the patient received PSL 40 mg and TAC 5 mg. On the basis of the analysis of cerebrospinal fluid (CSF) and findings from head magnetic resonance imaging (MRI), the patient was diagnosed with limbic encephalitis, a condition associated with profound brain dysfunction. Although viral involvement was suspected, definitive confirmation could not be established. Notably, this was the sole serious adverse event reported in BRT patients.

Cytomegalovirus (CMV) reactivation was monitored regularly. Out of 14 patients, 11 experienced reactivation. This included 5 out of 7 patients in the BRT group and 6 out of 7 patients in the TC group. All affected patients received appropriate treatment and achieved recovery. One patient in the BRT group recovered without experiencing CMV reactivation due to prophylactic administration of acyclovir.

### Gene expression differences observed before and after treatment are more closely linked to prognosis than to the specific treatment modality

**Figure 1:**
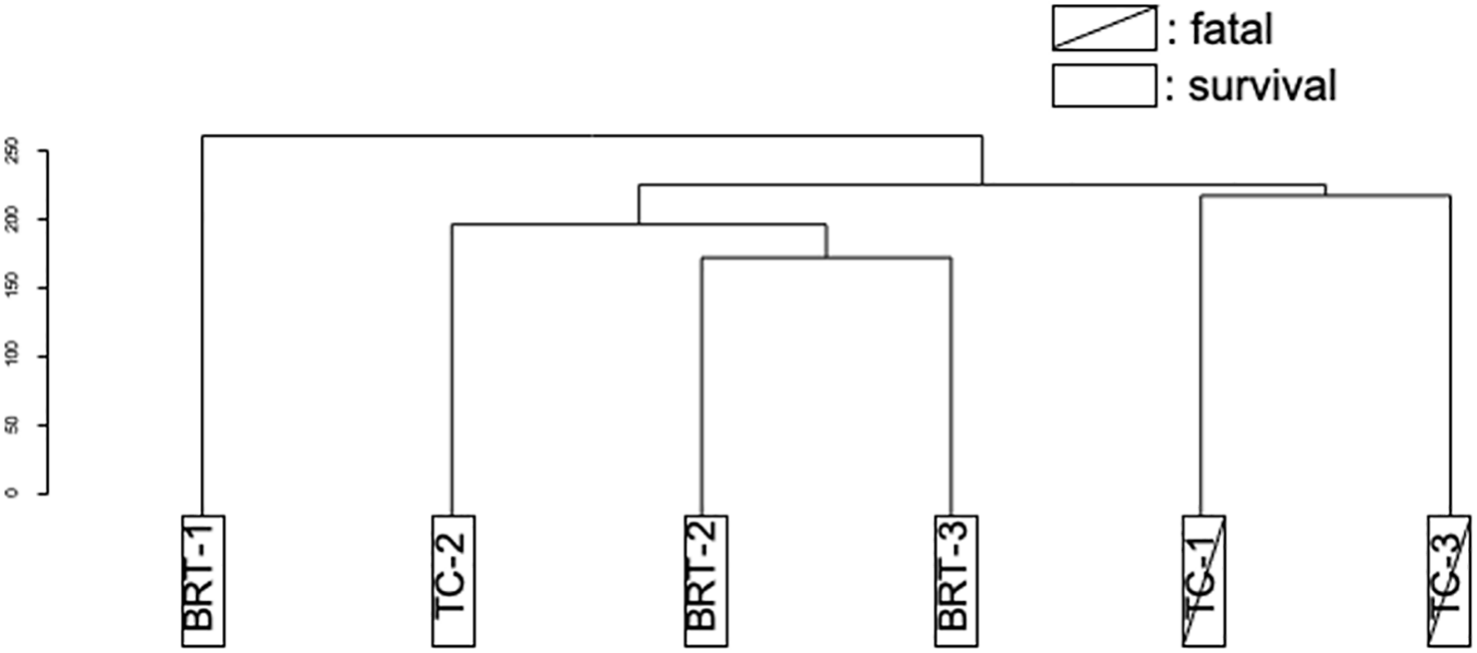
Clustering of DEGs (pre- *vs*. posttreatment). Cluster analysis of DEGs distinguished deceased patients from survivors, irrespective of the type of treatment administered.

Genetic analyses were conducted on the six patients (TC: n=3, BRT: n=3). DEGs identified before and after treatment were subjected to clustering analysis, which revealed that the DEGs did not stratify according to treatment group (TC *vs*. BRT) but instead aligned with patient outcomes (survival *vs*. mortality). These results suggest that variations in gene expression before and after treatment are not treatment dependent but are profoundly influenced by whether the patient survives or succumbs to the disease (**Figure 1**).

### Characteristics of gene expression changes following BRT and TC

To explore the distinctive characteristics of the effects of the TC and BRT therapies, GSVA and GO analyses were performed on the DEGs identified before and after treatment in the BRT (n = 3) and TC (n = 3) groups, respectively. GSVA revealed that, compared with TC-Tx, BRT-Tx significantly suppressed “leukocyte transendothelial migration” (p = 0.0198). Additionally, GO analysis of the genes that were downregulated after treatment among the DEGs revealed a significant reduction in the expression of genes related to B-cell functions in the BRT group, including those involved in “lymphocyte/B-cell proliferation” and the “B-cell receptor signaling pathway” (**Table 3A**). In contrast, GO analysis of the TC group indicated significant suppression of genes associated with nonspecific cellular processes, such as “cell proliferation” and “cell surface receptor signaling pathways” (**Table 3B**).

**Table 3:** GO analysis of DEGs (suppressed genes) in the BRT (n=3) and TC (n=3) groups.

(A) BRT group		
GO_IDs	Term	p-value
GO:0005886	plasma membrane	4.96E-08
GO:0071944	cell periphery	1.34E-07
GO:0046651	lymphocyte proliferation	2.11E-06
GO:0032943	mononuclear cell proliferation	2.28E-06
GO:0044459	plasma membrane part	2.41E-06
GO:0007166	cell surface receptor signaling pathway	4.90E-06
GO:0070661	leukocyte proliferation	4.98E-06
GO:0050853	B-cell receptor signaling pathway	6.10E-06
GO:0006959	humoral immune response	6.39E-06
GO:0009986	cell surface	9.40E-06
GO:0044425	membrane part	1.12E-05
GO:0042100	B-cell proliferation	1.14E-05
GO:0019814	immunoglobulin complex	1.91E-05
GO:0016021	integral component of membrane	2.62E-05
GO:0005887	integral component of plasma membrane	3.64E-05
GO:0031224	intrinsic component of membrane	3.78E-05
GO:0005261	cation channel activity	5.48E-05
(B) TC group		
GO:0044459	plasma membrane part	5.60E-11
GO:0031226	intrinsic component of plasma membrane	3.71E-10
GO:0005887	integral component of plasma membrane	1.99E-09
GO:0008283	cell proliferation	2.07E-09
GO:0042127	regulation of cell proliferation	2.08E-08
GO:0005886	plasma membrane	3.43E-08
GO:0007166	cell surface receptor signaling pathway	4.94E-08
GO:0071944	cell periphery	5.55E-08
GO:0051239	regulation of multicellular organismal process	6.84E-08
GO:0048731	system development	1.45E-07
GO:0007275	multicellular organism development	2.77E-07
GO:0008285	negative regulation of cell proliferation	7.84E-07
GO:0021648	vestibulocochlear nerve morphogenesis	8.14E-07
GO:0048584	positive regulation of response to stimulus	1.42E-06
GO:0061061	muscle structure development	2.82E-06
GO:0048856	anatomical structure development	3.34E-06
GO:0023052	signaling	3.95E-06

(A) The expression of B-cell-related genes, such as those involved in lymphocyte proliferation and the B-cell receptor signaling pathway, was significantly suppressed in the BRT group.

(B) TC therapy significantly suppressed pathways such as cell proliferation and cell surface receptor signaling pathways and was less specific for the target cells than was BRT.

### Characteristics of the surviving and fatal patients

GO analyses were conducted on the DEGs identified before and after treatment in surviving patients (n = 4) and fatal patients (n = 2). These analyses revealed significant suppression of the expression of B-cell-related genes, including those associated with the “immunoglobulin complex,” “B-cell receptor signaling pathway,” and “immunoglobulin receptor binding” (**Table 4A**). In our prior analysis involving a smaller cohort (12), the type I and II IFN signaling and RIG-I-like receptor pathways were ranked among the most significantly downregulated GO terms in survivors (n = 3) posttreatment. The lack of such findings in the current study appears to result from the inclusion of one additional survivor (BRT-3) whose IFN-related gene expression changes did not exceed the DEG threshold (|log_2_-fold change| ≥ 1).

**Table 4:** Characteristics of surviving patients (n=4) and fatal patients (n=2) according to GO analysis.

(A) Survival cases (downregulated genes)		
GO_IDs	Term	P-value
GO:0042571	immunoglobulin complex, circulating	2.83E-07
GO:0050853	B-cell receptor signaling pathway	1.65E-06
GO:0019814	immunoglobulin complex	9.73E-06
GO:0034987	immunoglobulin receptor binding	1.55E-05
GO:0006959	humoral immune response	3.70E-05
GO:0098886	modification of dendritic spine	4.35E-05
GO:0044459	plasma membrane part	5.62E-05
GO:0005886	plasma membrane	0.00033
GO:0050855	regulation of B-cell receptor signaling pathway	0.00046
GO:0006688	glycosphingolipid biosynthetic process	0.00059
GO:0071944	cell periphery	0.0006
GO:0005887	integral component of plasma membrane	0.00064
GO:0006682	galactosylceramide biosynthetic process	0.00064
GO:0019375	galactolipid biosynthetic process	0.00064
GO:0043539	protein serine/threonine kinase activator activity	0.00066
GO:0044425	membrane part	0.0007
GO:0061061	muscle structure development	0.00075
(B) Fatal cases (upregulated genes)		
GO:0005539	glycosaminoglycan binding	1.70E-05
GO:0035295	tube development	2.88E-05
GO:0042487	regulation of odontogenesis of dentincontaining tooth	4.80E-05
GO:0008201	heparin binding	8.73E-05
GO:0097477	lateral motor column neuron migration	0.000112
GO:0060128	corticotropin hormone secreting cell differentiation	0.000112
GO:0016151	nickel cation binding	0.000112
GO:0007267	cell-cell signaling	0.000123
GO:2000027	regulation of animal organ morphogenesis	0.00016
GO:0048513	animal organ development	0.000164
GO:2000648	positive regulation of stem cell proliferation	0.000198
GO:0042481	regulation of odontogenesis	0.000205
GO:0005102	signaling receptor binding	0.000221
GO:0097476	spinal cord motor neuron migration	0.000223
GO:0070326	very-low-density lipoprotein particle receptor binding	0.000223
GO:0001763	morphogenesis of a branching structure	0.000239
GO:0035239	tube morphogenesis	0.000245

GO analysis of DEGs (pre- *vs*. posttreatment) that were downregulated in surviving patients and upregulated in fatal patients. The immune system involving immunoglobulins and B cells was significantly suppressed in surviving patients.

Conversely, in fatal cases, the upregulation of genes associated with “glycosaminoglycan binding” and “heparin binding”—molecular functions implicated in the coagulation system—was observed (**Table 4B**).

### Comparison of IFN signatures before and after treatment

**Figure 2:**
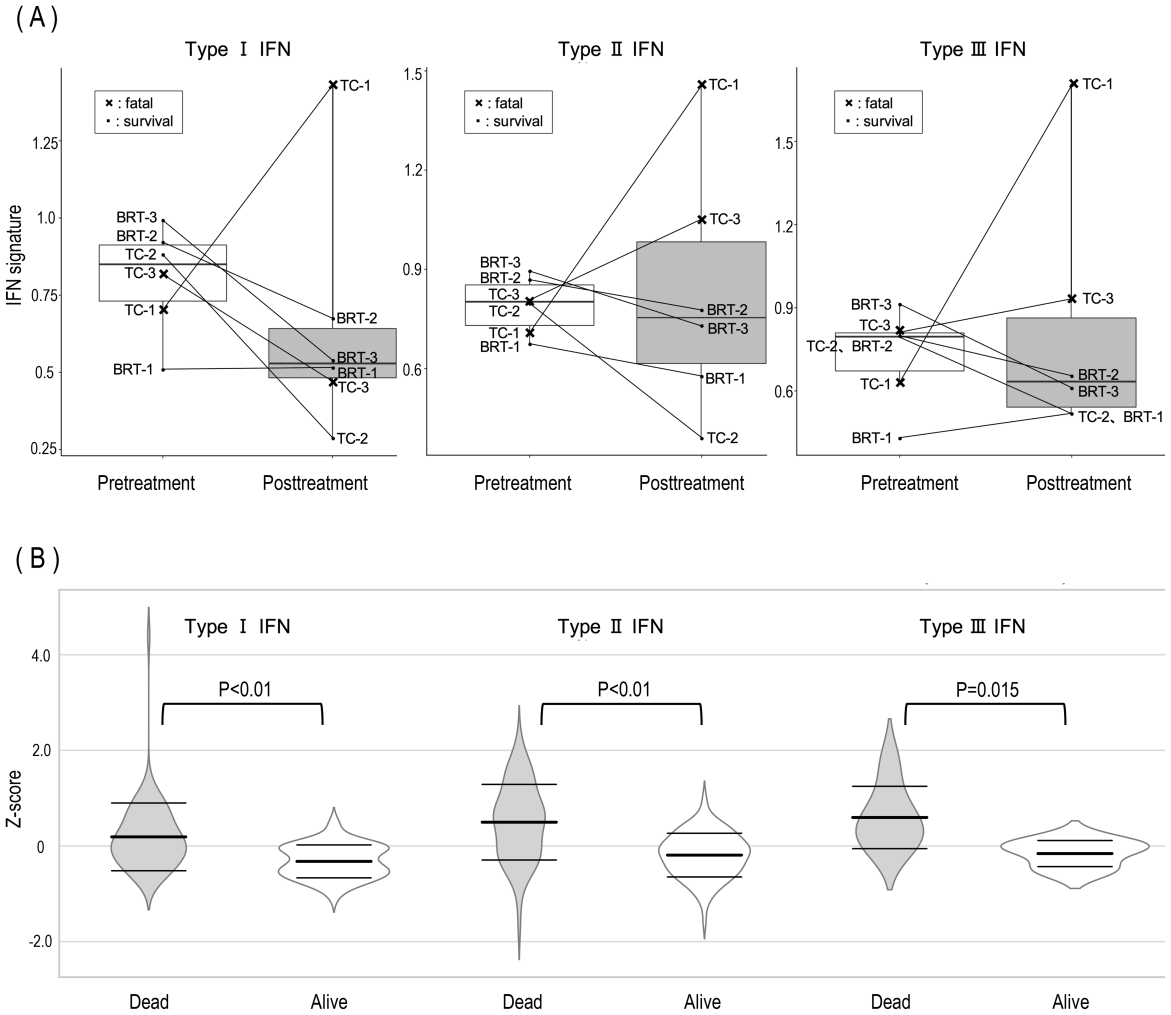
Posttreatment dynamics of IFN signature scores and gene expression in surviving and deceased MDA5-DM patients. **(A)** Posttreatment alterations in IFN signature scores revealed consistent increases in type II and type III IFN scores among all deceased patients, alongside an elevation in the type I IFN score in one deceased patient. The X symbols on the graph indicate deceased patients (TC-1 and TC-3). **(B)** Violin plots illustrating the distribution of log_2_ fold changes in type I, II, and III IFN-related gene expression between surviving and deceased patients. Central bold horizontal bars represent mean values, whereas upper and lower whiskers denote standard deviations. Statistical comparisons were performed via Welch’s t test.

Although the present GO analysis did not reveal significant suppression of type I interferon (IFN)-related genes in survivors, prior research has consistently implicated type I IFNs as key mediators in the pathogenesis of MDA5-DM (12, 33, 34). To investigate this further, IFN signature scores for type I, type II, and type III IFN pathways were calculated, and changes before and after treatment were analyzed for each patient (**Figure 2A**). When comparing BRT and TC therapies, no significant differences were observed in the trends of IFN signature scores before and after treatment. However, a distinct pattern was observed when surviving and deceased patients were compared. In surviving patients, the IFN signature score either decreased or remained low following treatment. In contrast, deceased patients presented increased IFN signature scores posttreatment. Notably, one of the two deceased patients presented an increase in type I IFN signatures, whereas both deceased patients presented elevated type II and type III IFN signatures after treatment. As shown in **Figure 2B**, the expression levels of type I, II, and III interferon-related genes before and after treatment were normalized via genewise Z scores. The distribution of expression changes was illustrated via violin plots. Notably, for all interferon types, gene expression was significantly lower in surviving patients than in deceased patients.

## Discussion

MDA5-DM is a rare autoimmune disease with high mortality rates, particularly when associated with ILD. Among these factors, RP-ILD is a well-recognized poor prognostic factor. A recent study reported various immunological abnormalities in active MDA5-DM patients, including hyperactivation of antibody-producing cells, CD8+ T-cell activation, and elevated levels of type I and II IFNs (33). While macrophages were not a primary focus of these studies, our autopsy findings suggest that in patients who succumb to RP-ILD associated with MDA5-DM, the majority of immune cells present in the alveoli are macrophages, implying that their dysregulation might represent a central pathological mechanism. The known correlation between elevated ferritin levels and macrophage activation (35), as well as the recognition of hyperferritinemia as a critical marker of disease activity and poor prognosis (15), further support this hypothesis.

The management of severe anti-MDA5 antibody-positive dermatomyositis (MDA5-DM) remains challenging, with no standardized therapy established yet. The rarity and rapid progression of this disease limit the feasibility of randomized controlled trials, thereby restricting high-level evidence. Studies have shown that TC-Tx, a combination of tacrolimus (TAC), cyclophosphamide (CYC) and high-dose glucocorticoids (GCs), is effective in newly diagnosed MDA5-DM patients with ILD (20, 36). Consequently, TC-Tx is widely used initially, especially for rapidly progressive ILD (RP-ILD) (37). However, some cases remain refractory.

Mycophenolate mofetil (MMF), an immunosuppressant that inhibits DNA replication in lymphocytes via inosine monophosphate dehydrogenase, has shown efficacy in refractory cases of MDA5-DM with ILD (38). Alternative combinations using MMF instead of CYC show potential, although evidence is currently limited.

The Janus kinase (JAK) inhibitor tofacitinib (TOF), the first approved JAK inhibitor, which is commonly used in MDA5-DM, has shown benefits in refractory cases (39). Reports on other JAK inhibitors are sparse. Additionally, the biologic agent rituximab (RTX) has shown promise, with survival benefits reported in combination with TOF and plasma exchange (PE) (40). Further research is needed to confirm these therapeutic options.

This study investigated the efficacy and underlying mechanisms of a novel multitarget combination therapy, BRT-Tx (baricitinib [BAR], rituximab [RTX], tacrolimus [TAC], and high-dose glucocorticoids [GCs]), in seven patients with MDA5-DM-associated ILD, including patients who exhibited rapid progression. These findings were compared with those of seven historical patients treated with TC-Tx (tacrolimus [TAC], cyclophosphamide [CYC], and high-dose glucocorticoids [GCs]).

Our findings demonstrated the superiority of BRT-Tx over TC-Tx in improving survival outcomes. All seven patients treated with BRT-Tx survived, and only one required combined rescue therapy to manage disease relapse. In contrast, four of the seven TC-Tx cases resulted in fatal outcomes, underscoring the limitations of conventional regimens.

Transcriptome analyses further highlighted the mechanistic differences between the therapies: BRT-Tx effectively suppressed lymphocyte-related genes, including those involved in the B-cell receptor signaling pathway, whereas TC-Tx predominantly suppressed genes associated with nonspecific cell proliferation.

Gene ontology analysis comparing survivors and nonsurvivors revealed that excessive B-cell activation and antibody-mediated effector responses were suppressed in the surviving group. Although IFN-related GO terms were not enriched, this was likely due to the modest expression changes in many IFN-related genes, which did not meet the DEG threshold (|log_2_-fold change| ≥ 1). When focusing specifically on IFN-related genes, posttreatment expression of the type I, II, and III IFN pathways was significantly downregulated in survivors. These findings underscore the pivotal role of suppressing B-cell–driven signaling and further suggest that attenuation of interferon responses may also contribute to favorable therapeutic outcomes in MDA5-DM patients.

These results suggest that BRT-Tx provides a targeted approach to modulating immune dysregulation in MDA5-DM patients, offering promising outcomes even in high-risk patients with poor prognostic factors.

### Mechanisms of BRT therapy

BRT-Tx was developed as a multitarget therapeutic regimen designed to address the complex immunopathology of MDA5-DM. Each component was selected for its ability to modulate specific immune pathways:

1. Baricitinib (BAR): Suppressing the JAK-STAT pathway.

BAR, a JAK1/2 inhibitor, suppresses type I and II IFN signaling and the granulocyte–macrophage colony–stimulating factor (GM–CSF) pathway. These pathways are critical for macrophage activation and inflammatory cascades in the pathogenesis of MDA5-DM. Compared with tofacitinib (TOF), BAR more strongly inhibits JAK2, resulting in superior suppression of GM-CSF and IFN-γ signaling (41). Furthermore, the antifibrotic properties of BAR, as demonstrated in a murine model of bleomycin-induced pulmonary fibrosis (42), suggest its potential to reduce lung injury and fibrosis in human diseases such as MDA5-DM-associated ILD. Its clinical efficacy in severe COVID-19 pneumonia, which shares pathological features with MDA5-DM, further supports its use (43, 44). On the other hand, it suppresses the IFN signaling pathway, necessitating heightened vigilance to prevent the reactivation of latent viral infections. Particularly during the period of BAR administration, regular monitoring for CMV reactivation is essential, with assessments recommended at least once every 1–2 weeks, and prophylactic administration of acyclovir is also recommended.

2. Rituximab (RTX): Targeting B cells and autoantibody production.

RTX offers a targeted approach to suppressing B-cell-mediated immune responses by depleting CD20-positive B cells, ultimately leading to a reduction in the production of anti-MDA5 autoantibodies. This mechanism is particularly important in managing MDA5-DM, where autoantibody titers are strongly correlated with disease prognosis (14).

Compared with cyclophosphamide (CYC), RTX is a safer alternative. CYC is effective in reducing autoantibody production but is associated with significant long-term risks, including infertility and carcinogenesis (45), making its use less favorable, particularly in younger patients. Additionally, RTX has demonstrated the ability to deplete B cells rapidly, providing earlier modulation of immune responses than CYC does.

The safety profile of RTX is further supported by studies indicating fewer adverse events than CYC in the treatment of collagen disease-associated ILD while maintaining comparable efficacy in controlling disease activity (46). These characteristics position RTX as a preferred option for patients with MDA5-DM and poor prognostic factors, where rapid and targeted immunomodulation is critical.

3. Tacrolimus (TAC): Suppressing T-cell activation.

TAC inhibits calcineurin-mediated T-cell activation, addressing the hyperactivated CD8+ T cells implicated in MDA5-DM pathogenesis (20, 33). Its established safety and efficacy in autoimmune diseases complement the actions of BAR and RTX, ensuring comprehensive immune modulation.

### Prophylactic management of opportunistic infections

Potent immunosuppressive therapies inherently increase susceptibility to opportunistic infections, which can lead to life-threatening complications if not adequately addressed. Accordingly, rigorous infection prophylaxis is essential. To mitigate the risk of *Pneumocystis jirovecii* pneumonia (PCP) and fungal infections, sulfamethoxazole-trimethoprim (ST combination) and antifungal agents—such as oral amphotericin B suspension—are routinely administered to high-risk patients receiving intensive immunosuppression.

In the context of the BRT-Tx regimen, baricitinib (BAR), a JAK inhibitor, suppresses IFN signaling and consequently elevates the risk of reactivation of latent viral infection. Prophylactic antiviral administration and close monitoring are thus warranted. Acyclovir is widely used for its established efficacy against herpes simplex virus types 1 and 2 (HSV-1/2), varicella-zoster virus (VZV), and Epstein–Barr virus (EBV) and has also been reported to exhibit modest activity against cytomegalovirus (CMV). Given the relatively high incidence of CMV reactivation during immunosuppressive therapy, regular virological monitoring is indispensable. In patients receiving BAR, CMV DNAemia/antigenemia should be assessed at intervals of 1 to 2 weeks. Although ganciclovir possesses greater intrinsic activity against CMV, acyclovir is selected for prophylaxis because CMV reactivation can be readily surveilled and treated preemptively, whereas HSV reactivation is difficult to detect and, on occasion, culminates in severe encephalitis.

### Combined rescue therapy

One patient who underwent BRT-Tx required combined rescue therapy, which included high-dose glucocorticoids (GCs), plasma exchange (PE), and intravenous immunoglobulin (IVIG), to manage disease relapse. PE facilitates a rapid reduction in circulating autoantibody levels, thereby creating a critical window for RTX-induced B-cell depletion to exert therapeutic effects (25, 26). In the present study, antibody-mediated effector responses were significantly suppressed in the survivor group, suggesting that PE-mediated autoantibody clearance may represent a useful adjunctive strategy in the treatment of MDA5-positive dermatomyositis. Additionally, IVIG contributes to the modulation of excessive immune responses, further stabilizing the patient’s condition (47, 48). This case highlights that while BRT-Tx may not serve as a universal solution for all MDA5-DM patients, it holds significant potential to achieve life-saving outcomes when strategically combined with adjunctive therapies.

The protocol and management considerations for BRT therapy are listed in **Table 1**.

### Limitations and future directions

The small sample size and retrospective nature of this study impose limitations on the generalizability of its findings. Moreover, while there was no significant difference in the number of poor prognostic factors among the patients, variability in the severity of ILD at the initiation of treatment introduces a potential confounding factor that may influence the interpretation of survival outcomes. To address these limitations, a randomized controlled trial (RCT) comparing BRT-Tx and TC-Tx in MDA5-DM patients with poor prognostic factors is planned. This trial aims to provide more robust and definitive evidence to substantiate the findings of this study.

## Conclusions

This study highlights the superiority of BRT-Tx over historical TC-Tx in managing MDA5-DM-associated ILD. By targeting key immune pathways through BAR, RTX, and TAC, BRT-Tx offers a comprehensive and effective approach for controlling disease progression, even in high-risk patients. These findings support the inclusion of BRT-Tx as a first-line therapeutic option for MDA5-DM patients with multiple poor prognostic factors, warranting further validation in larger, prospective trials.

## Data Availability

The datasets presented in this study can be found in online repositories. The names of the repository/repositories and accession number(s) can be found below: https://www.ddbj.nig.ac.jp/, DRA020447.
